# Cepharanthine Alleviates DSS-Induced Ulcerative Colitis via Regulating Aconitate Decarboxylase 1 Expression and Macrophage Infiltration

**DOI:** 10.3390/molecules28031060

**Published:** 2023-01-20

**Authors:** Min-Na Zhang, Rui Xie, Hong-Gang Wang, Xin Wen, Jing-Yi Wang, Le He, Meng-Hui Zhang, Xiao-Zhong Yang

**Affiliations:** 1 Department of Gastroenterology, The Affiliated Huai’an No.1 People’s Hospital of Nanjing Medical University, Huai’an 223300, China; 2 Digestive Disease Center, The Affiliated Huai’an No.1 People’s Hospital of Nanjing Medical University, Huai’an 223300, China

**Keywords:** Cepharanthine, gut microbiota, colitis, ACOD1, macrophage

## Abstract

Cepharanthine (CEP), a bisbenzylisoquinoline alkaloid from tubers of Stephania, protects against some inflammatory diseases. Aconitate decarboxylase 1 (ACOD1) is also known as immune-responsive gene 1 (IRG1), which plays an important immunometabolism role in inflammatory diseases by mediating the production of itaconic acid. ACOD1 exhibits abnormal expression in ulcerative colitis (UC). However, whether CEP can combat UC by affecting ACOD1 expression remains unanswered. This study was designed to explore the protective effects and mechanisms of CEP in treating colitis through in vitro and in vivo experiments. In vitro assays indicated that CEP inhibited LPS-induced secretion of pro-inflammatory cytokines and ACOD1 expression in RAW264.7 macrophages. Additionally, in the mouse model of DSS-induced colitis, CEP decreased macrophage infiltration and ACOD1 expression in colon tissue. After treatment with antibiotics (Abx), the expression of ACOD1 changed with the composition of gut microbiota. Correlation analysis also revealed that Family-XIII-AD3011-group and Rumini-clostridium-6 were positively correlated with ACOD1 expression level. Additionally, data of the integrative Human Microbiome Project (iHMP) showed that ACOD1 was highly expressed in the colon tissue of UC patients and this expression was positively correlated with the severity of intestinal inflammation. Collectively, CEP can counter UC by modulating gut microbiota and inhibiting the expression of ACOD1. CEP may serve as a potential pharmaceutical candidate in the treatment of UC.

## 1. Introduction

Ulcerative colitis (UC), a major subtype of inflammatory bowel disease (IBD), arises from chronic and recurrent inflammation in the colonic mucosa and submucosa, clinically manifested as persistent or recurrent diarrhea, mucus pus, and bloody stool with abdominal pain, tenesmus, and various degrees of systemic symptoms [1]. Over the past decade, the prevalence of UC has increased dramatically, making its treatment a great challenge worldwide, especially in China where the first case was reported in 1956 [2]. UC tends to occur in the population at a younger age, globally [3]. Novel effective therapeutic options are urgently needed to cope with this global threat.

IBD involves various pathogenic factors, including environmental assaults, immune activation, genetic susceptibility, and intestinal microbiota [4]. Notably, immune cells play an essential role [5]. Among them, macrophages regulate the immune response when the intestinal mucosal barrier is damaged [6]. Thus, macrophages may be targeted to create new treatment strategies for IBD [7]. Additionally, the role of gut microbiota in the pathogenesis of IBD has attracted critical attention [8,9]. Gut microbiota plays a high profile in maintaining immune homeostasis [10]. Some traditional Chinese medicine (TCM) formulae have shown benefits in the treatment of IBD by regulating gut microbiota [11]. These data provide clues of employing TCM to treat UC [12].

*Fangchiaceae*, as a well-known group of medicinal plants, have shown antipyretic, analgesic, and anti-inflammatory properties in clinical practice [13]. Anti-inflammatory ingredients in *Fangchiaceae* include Dauricine (DA), Jatrorrhizine, Magnoflorine (MA), and Cepharanthine (CEP). DA counters inflammation in LPS/CLP-induced acute lung injury (ALL) through inhibiting the activation of NF-κB and the production of pro-inflammatory cytokines [14]. Jatrorrhizine (JA) downregulates pro-inflammatory cytokines to curb the development of RA, and its mechanism involves the attenuation of NF-κB and MAPK stimulated by tumor necrosis factor TNF-α [15]. Magnolia (MA) can reduce NP cell damage mediated by M1-polarized macrophages by inactivating the HGB1-MyD88-NF-κB pathway and the NLPR3 inflammasome [16]. For more than 40 years, CEP has been used to treat inflammatory diseases, such as rheumatism, lumbago, nephritis edema, and dysentery [17]. CEP can suppress the activation of macrophages to reduce the release of inflammatory factors, such as TNFα, IL-1β, and IL-6 [18]. However, the efficacy of CEP in the treatment of ulcerative colitis has not been analyzed.

Aconitate decarboxylase 1 (ACOD1), also known as immune responsive gene 1(IRG1), acts on the tricarboxylic acid intermediate product cis-aconitic acid, and decarboxylates it into itaconic acid. ACOD1 and its products have linked alterations in cellular metabolism to immune defense, and mediated immune-regulatory responses through multiple pathways [19]. Studies have shown that ACOD1 is observed to be highly expressed in LPS-and IFNG-activated macrophage mitochondria in a pro-inflammatory state, blocking the tricarboxylic acid cycle [20]. ACOD-1 mediated itaconate production has shown the regulation of inflammation in various preclinical models, including pulmonary fibrosis [21], liver ischemia–reperfusion injury [22], and pulmonary Brucella infection [23]. The role of the ACOD1-clathrate axis in colitis was first proposed in the study by Kim et al. Therefore, we speculate that ACOD1 may play an important regulatory role in the pathogenesis of colitis [24].

In this study, we analyzed the efficacy and mechanism of CEP in treating DSS-induced colitis using in vitro and in vivo experiments. The role of microbiota and ACOD1 expression in this mechanism was also elucidated.

## 2. Result

### 2.1. CEP Protects the Mice from DSS-Induced UC

**Figure 1:**
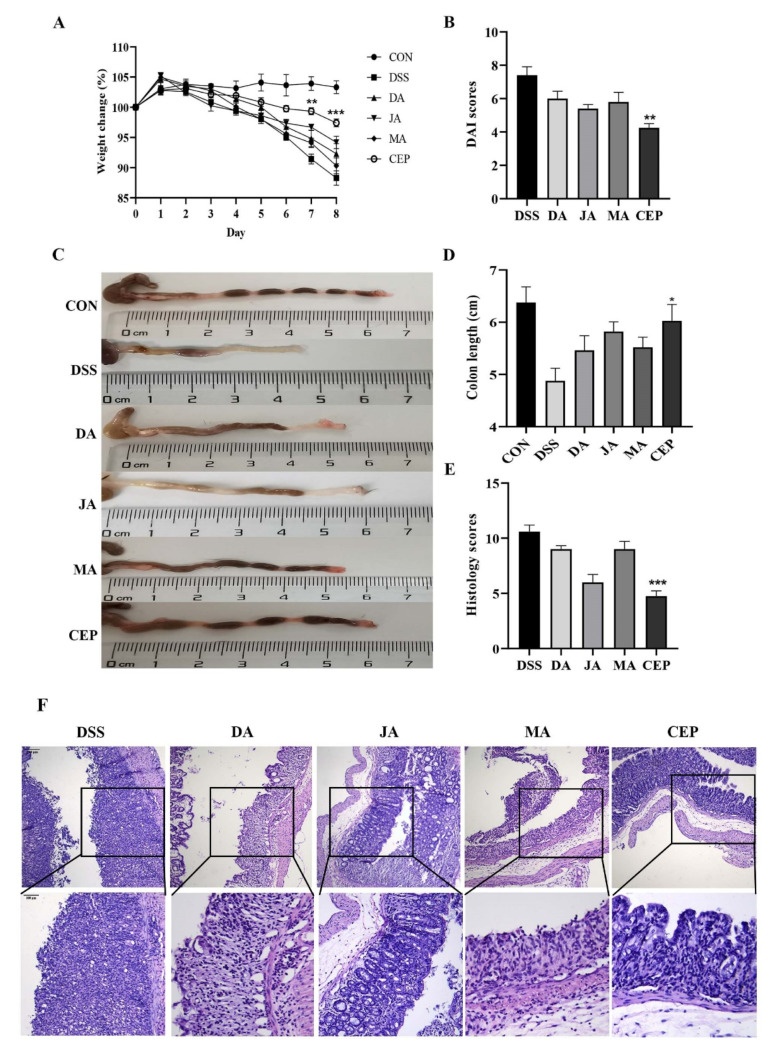
CEP protects the mice from DSS-induced UC. (**A**) The percentage of body weight changes of mice during drug administration; The* represents significant difference between the DSS and CEP group and (**B**) the DAI score; (**C**) representative photographs of the colon; (**D**) the colon length; (**E**) the histology score; values are presented as the mean ± standard error of the mean (SEM); n = 5 in each group; * *p* < 0.05, ** *p* < 0.01, *** p < 0.001. (**F**) H&E staining of mice colon tissue, the photos were observed by confocal laser-scanning microscope, 100×, 200×.

A significant decrease was observed in the bodyweight of mice after 7 days of DSS administration. After treatment with four ingredients from *Fangchiaceae*, the bodyweight recovered to a certain extent, most obviously in the CEP-treated group (Figure 1A). The DAI score showed that the severity of intestinal inflammation decreased significantly in the CEP-treated group, compared with other treatment groups (Figure 1B). The length of the shortened colon could serve as an objective indicator to reflect the degree of intestinal inflammation. As expected, CEP restored the colon length to a degree in UC mice (Figure 1C). H&E staining showed the loss of colonic epithelial cells, the disruption of crypt structure, and massive infiltration of inflammatory cells in DSS-treated mice (Figure 1D). After treatment with four drugs, the colonic inflammation was relieved, as shown by the relatively intact colon epithelium and remarkably decreased inflammatory cells. The histological score confirmed that CEP was the most effective in improving intestinal inflammation in mice (Figure 1E).

### 2.2. CEP Inhibits LPS-Induced Secretion of Pro-Inflammatory Cytokines and ACOD1 Expression in RAW264.7 Macrophages

**Figure 2:**
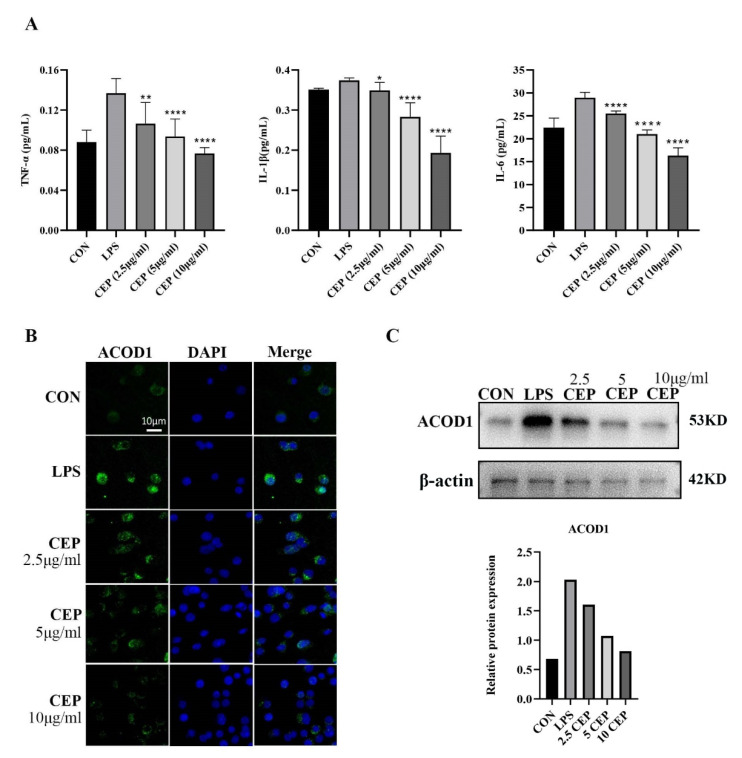
CEP inhibits LPS-induced secretion of pro-inflammatory cytokines and ACOD1 expression in RAW264.7 macrophages. (**A**) Concentrations of inflammatory factors (TNF-α, IL-1β, and IL-6) in RAW264.7 cell culture medium were measured by ELISA after different concentrations of CEP and LPS administration for 24 h; (**B**) ACOD1 detection in macrophages by immunofluorescence microscopy; (**C**) ACOD1 detection in macrophages by Western blot (WB). * *p* < 0.05; ** *p* < 0.01; **** *p* < 0.001.

RAW264.7 cells were used to determine the in vitro anti-inflammatory effects of CEP. We examined pro-inflammatory cytokine levels in RAW264.7 macrophages exposed to various concentrations of CEP in the presence of LPS by qRT-PCR. The results showed that the levels of TNF-α, IL-1β, and IL-6 were reduced in the supernatant after exposure to different concentrations of CEP, most evidently to 10 ug/mL CEP (Figure 2A). Cell RNA sequencing results suggested that the expression of ACOD1 in macrophages increased most significantly after LPS stimulation (Table 1). Subsequently, we used LPS-activated macrophages as a positive control, and macrophages were treated with CEP of various concentrations for 24 h. Immunofluorescence (IF) staining revealed that as the CEP concentration increased, the expression of ACOD1 dropped in macrophages. Especially, 10 ug/mL CEP exhibited the most significant inhibitory effect (Figure 2B). WB also verified a similar result at the protein level (Figure 2C). These results indicated that CEP exerted an anti-inflammatory effect by inhibiting macrophage-released pro-inflammatory factors and expression of ACOD1.

### 2.3. CEP Decreases the Infiltration of Macrophages and ACOD1 Expression in the Mice with DSS-Induced UC

**Figure 3:**
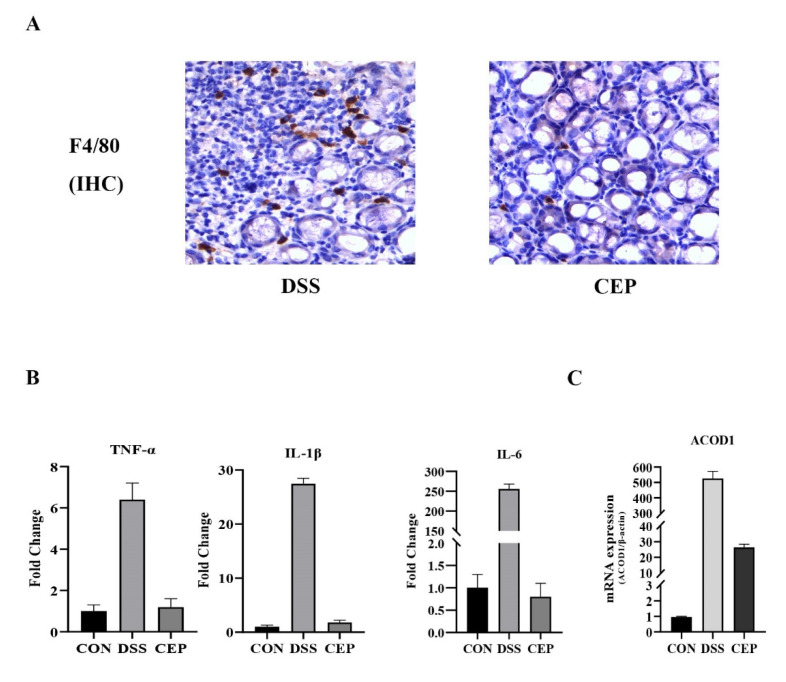
CEP decreases the infiltration of macrophages and ACOD1 expression in DSS-induced colitis. (**A**) Infiltrated macrophages in colon tissue were detected by immunohistochemical staining of F4/80. (**B**) Transcriptome sequencing analysis of inflammatory factors TNF-α, IL-1β, and IL-6 transcript levels in mouse colon tissue (Both *p* values <0.001); (**C**) ACOD1 mRNA expression in colon tissue measured by qRT- PCR (*p* values <0.001).

F4/80, a major macrophage marker, was detected to assess the macrophage infiltration in colon tissue. IHC staining revealed that F4/80+ macrophages increased obviously in the DSS group, and decreased after CEP administration (Figure 3A). Transcriptome sequencing was performed to identify DEGs between CEP-treated and DSS-induced groups. The transcription levels of proinflammatory cytokines (TNF-α, IL-1β, IL-6) in the CEP-treated group were significantly lower than those in the DSS-induced group (Figure 3B). The top ten DEGs were identified (Table 1). Consistent with the experimental data of RAW264.7 macrophages, the expression of ACOD1 was also significantly different at the transcription level in vivo. CEP effectively decreased the transcription level of ACOD1 (Figure 3C). Together, CEP could ameliorate colonic inflammation by reducing macrophage infiltration and the expression of ACOD1.

### 2.4. CEP Acts on Gut Microbiota to Regulate ACOD1 Expression

**Figure 4:**
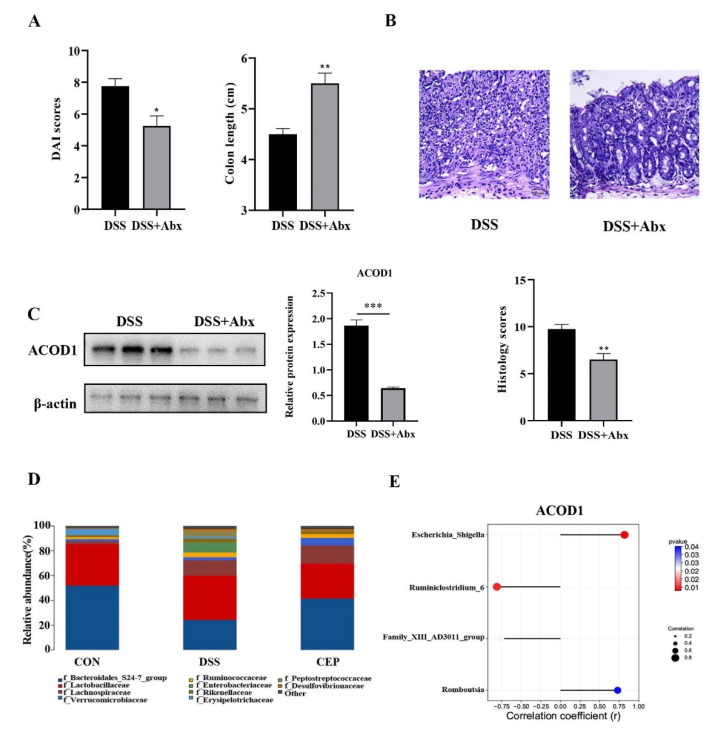
CEP acts on gut microbiota to regulate ACOD1 expression. (**A**) DAI score between the DSS group and DSS+ABX group; (**B**) H&E staining of mice colon tissue (X100); the histology score; (**C**) the protein expression ACOD1 assessed by WB; the histology score; (**D**) relative abundance of gut microbiota at the family levels; (**E**) correlation analysis between ACOD1 and gut microbiota. * *p* < 0.05; ** *p* < 0.01; *** *p* < 0.001.

We further explored other factors that affect the expression of ACOD1. We destroyed the gut microbiota of C57BL/10 mice by pretreating with broad-spectrum Antibiotics (Abx) (vancomycin, colistin, neomycin, and metronidazole), and the results showed the alleviation of DSS-induced colonic inflammation. After Abx pretreatment, the mice showed a lower DAI score and a longer colon than the DSS group (Figure 4A). H&E staining also confirmed the reduction in intestinal inflammation in the mice after Abx pretreatment, with a better structure of intestinal epithelium and crypts, and less infiltration of inflammatory cells (Figure 4B). This change was also confirmed by the pathological scores. We also found that the protein level of ACOD1 expression was also decreased with the reduction in intestinal inflammation after Abx treatment (Figure 4C). The effect of Abx on gut microbiota was analyzed using 16S rRNA sequencing. At the family level, *Peptostreptococcaceae* and *Enterobacteriaceae* were more abundant in the DSS group than in the control group (*p* < 0.05), whereas CEP reversed their levels close to those in the control group (Figure 4D). In addition, it has been shown in our previous study that CEP can remarkably decrease the abundance of *Escherichia-Shigella* and *Romboutsia* after DSS administration. The relative abundances of *Family-XIII-AD3011-group* and *Ruminiclosridium-*6 were reduced by DSS, but further restored by CEP [25]. Attached to this, we analyzed the association between these bacterial genera and ACOD1 (Figure 4E). The abundances of *Escherichia-Shigella* and *Romboutsia* were found to be negatively correlated with ACOD1 expression level, while the abundances of *Family-XIII-AD3011-group* and *Ruminiclostridium-6* were positively correlated with ACOD1 expression level. Both correlations were statistically significant. In summary, gut microbiota were involved in CEP-regulated ACOD1 expression.

### 2.5. ACOD1 Is Highly Expressed in the Colon Tissue of UC Patients and Associated with Intestinal Inflammation

**Figure 5:**
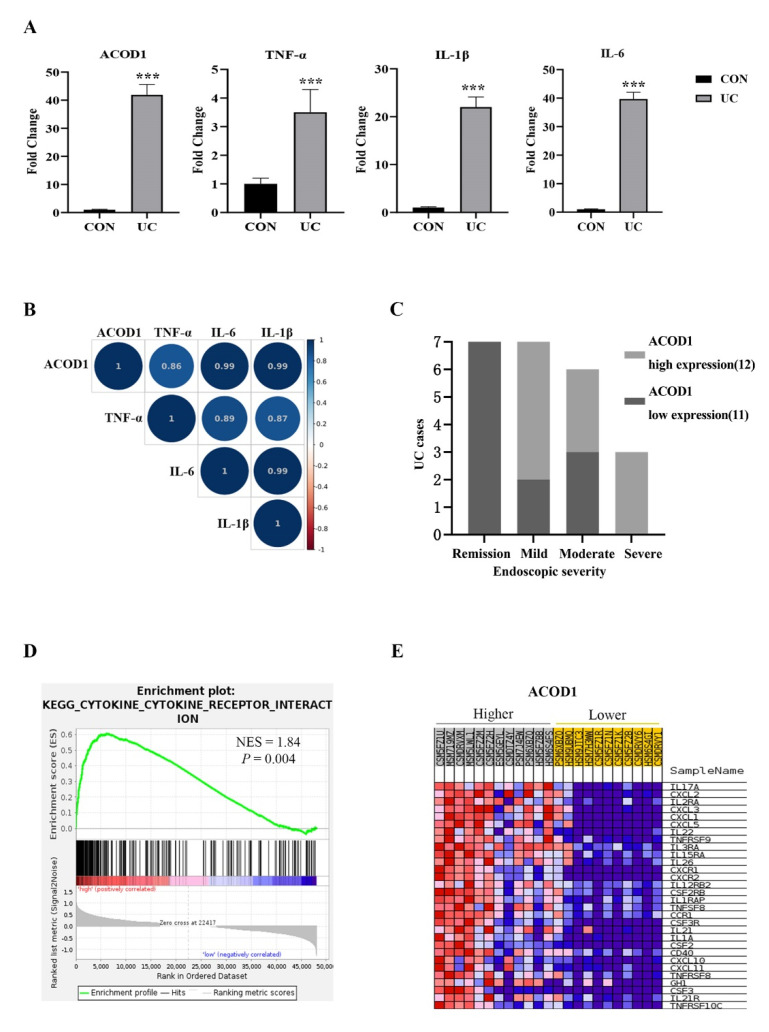
ACOD1 is highly expressed in the colon tissue of UC patients and associated with intestinal inflammation. (**A**) The transcription levels of inflammatory factors (TNF-α, IL-1β, and IL-6), and ACOD1 in UC patients; (**B**) correlation of ACOD1 with the transcription levels of inflammatory factors TNF-α, IL-1β, and IL-6 (*p* < 0.001); (**C**) correlation of ACOD1 transcript levels with disease severity in UC patients; (**D**) the enrichment plot from gene set enrichment analysis (GSEA); (**E**) top 30 ACOD1 significantly associated genes. *** *p* < 0.001.

Based on the above experimental results, we speculated that ACOD1 may play an essential role in intestinal inflammation. We excavated the IBD database (IBD MDB) in the Human Microbiome Project (iHMP), selected UC cases with complete omics data for research, and analyzed the ACOD1 transcription in the rectal mucosa of 23 UC patients and 20 non-IBD controls (control group). Compared with that in the control group, the transcription level of ACOD1 in the UC group increased by 42 times, and the transcription levels of TNF-α, IL-1β, and IL-6 also increased significantly (*p* < 0.01) (Figure 5A). Based on the median level of ACOD1 transcription, the 23 UC cases were divided into two groups: the high ACOD1 expression group (12 cases) and the low ACOD1 expression group (11 cases). Among the seven patients with UC in remission, none showed high expression of ACOD1. Among the three patients with severe UC, none showed low expression of ACOD1. The results suggested that ACOD1 was significantly associated with the severity of UC (Figure 5C). Additionally, the ACOD1 expression level was also significantly positively correlated with the transcription levels of intestinal inflammatory factors TNF-a, IL-1B, and IL-6 (*p* < 0.001) (Figure 5B). Next, gene set enrichment analysis (GSEA) discovered that the DEGs were most significantly enriched in signaling pathways related to cytokines and cytokine receptors, mainly interleukins, tumor necrosis factors, chemokines, and their receptors (Figure 5D,E). Together, ACOD1 was involved in the expression of inflammatory factors in the condition of UC.

## 3. Methods

### 3.1. Cell Culture

RAW 264.7 cells were purchased from American Type Culture Collection (ATCC) and cultured in DEME containing 10% fetal bovine serum (FBS; Hyclone) in a humidified atmosphere at 37 °C with 5% CO_2_ until 70–80% confluence (2–3 days) and 1:4 split.

### 3.2. ELISA

RAW 264.7 cells were seeded to 24-well plates and treated with different concentrations of CEP (2.5 ug/mL, 5 ug/mL, 10 ug/mL). Then, the cells were incubated for 24 h with or without 1 ug/mL LPS (Sigma, L2880). According to the manufacturer’s instructions, the concentrations of cytokines TNF-α, IL-6 and IL-1β were determined in the supernatant using Mouse-specific ELISA kits (Biolegend, San Diego, CA, USA).

### 3.3. Immunofluorescence Staining

RAW264.7 cells were allowed to grow on glass coverslips. After 24 h of treatment, the cells were fixed in 4% paraformaldehyde and permeabilized with 0.2% TritionX-100 in PBS for 20 min. Then, the slides were incubated with goat serum at room temperature for 30 min. ACOD1 (dilution 1:100; Abcam) primary antibodies were added to the sections and incubated at 4 °C overnight. Sections were soaked 3 times, for 3 min each time. The diluted fluorescent secondary antibodies were added, and the samples were incubated for 1 h at room temperature. Following counterstaining with DAPI for the nuclei, the cells were observed under a fluorescence microscope.

### 3.4. Animal Experiments

Female C57BL/10 (20–25 g) mice aged 7–8 weeks were purchased from the Model Animal Research Center of Nanjing University. All mice were maintained at the Center for Experimental Animal of Huai’an First People’s Hospital. The mice were acclimatized to the new environment at 21 ± 2 °C and 45 ± 10% humidity, with a 12 h light/12 h dark cycle. This study was approved by the Animal Ethics Committee of Nanjing Medical University. After one week of acclimation, the mice were randomly divided into (1) control group in which the mice were given gavage of DSS-free sterile distilled water on days 1 to 7; (2) DSS group in which the mice were given 2.5% (*w*/*v*) DSS in their drinking water for 7 days to induce acute colitis, as well as water gavage on days 1 to 7; (3) medication group in which the mice were given 2.5% DSS plus oral gavage of medications daily for 7 days (DA 20 mg/kg/d, JA 20 mg/kg/d, MA 20 mg/kg/d, MA and CEP 10 mg/kg/d). At the end of the experiment, the mice were euthanized, and fresh feces and colon tissues were collected for further analysis. DSS (molecular weight: 36–50 kDa) was purchased from MP Biomedicals (MP Biomedicals, USA). DA, JA, MA, and CEP were purchased from Chengdu Biopurify Phytochemicals (Sichuan, China).

### 3.5. Disease Activity Index (DAI)

The DAI score was calculated by summing the scores of body weight loss (1, 1–5%; 2, 5–10%; 3, 10–15%; and 4, ≥ 15%), stool consistency (0, normal; 2, loose stools; 4, diarrhea), and blood in the stool (0, no blood seen; 2, apparent blood with stool; 4, grossly bloody stool). A loss of more than 30% of body weight was used as a criterion for performing humane euthanasia to reduce the pain of the mice.

### 3.6. Histopathological Analysis

The distal colon tissue was obtained and fixed in 4% paraformaldehyde for histopathological analysis. Colon tissue samples were dehydrated in gradient alcohol and embedded in paraffin. Sections (4 μm) were stained with hematoxylin and eosin (H&E). The histological score was calculated according to the degrees of inflammation, crypt damage, lymphocytic infiltration, and colon wall integrity.

### 3.7. Immunohistochemical Staining

The mice colon tissue sections were dewaxed with xylene for 30 min, and sequentially in 100%, 95%, 80%, and 70% ethanol and ddH_2_O for 5 min. The slices were incubated in methanol containing 3% H_2_O_2_ for high-pressure antigen retrieval, and the repaired sections were sealed with 5% BSA for 30 min. F4/80+ (Abcam, Cambridge, MA, USA) primary antibodies were incubated overnight at 4 °C. After washing with PBS 3 times, the slices were incubated with appropriate secondary antibodies. DAB was used as the chromogen, and counterstaining was performed with hematoxylin. Finally, the sections were sealed with neutral resin and observed under an optical microscope.

### 3.8. Quantitative Real-Time PCR

Total RNA was extracted from cells and colon tissues by RNA simple Total RNA Kit (TIANGEN, DP419, Tianjin, China). Then, 2 μL of extraction was pipetted to measure the total RNA concentration by Nanodrop. RNA was then reversely transcribed into cDNA using a RevertAid First Strand cDNA Synthesis Kit (Thermo, #K1622, USA). Quantitative RT-PCR was performed using SYBR Green I (TsingKe, Beijing). The relative expression level of ACOD1 was normalized to that of DAPDH using the 2-ΔΔCt method. (Primer sequence, ACOD1, F: GGTATCATTCGGAGGAGCAA, R: ACAGAGGGAGGGTGGAATCT; β-actin, F: CCTGTGGCATCCATGAAACT, R: GTGCTAGGAGCCAGAGCAGT).

### 3.9. Western Blot

Cells or colon tissues were homogenized in RIPA lysis buffer with PMSF (RIPA:PMSF = 100:1, *v*/*v*). Afterward, total protein was extracted from the supernatant. The protein was quantified by the BCA Protein Assay Kit (Beyotime, Haimen, China). Then, the equal amount of protein was loaded and separated by 10% SDS-PAGE and transferred to the PVDF membrane. Membranes were blocked in 5% nonfat milk for 2 h and incubated with the primary antibodies at 4 °C overnight. Having been rinsed again in TBST 3 times, the membranes were incubated with the secondary antibodies on a shaker for 1 h at room temperature, then washed again 3 times with TBST (10 min each time). Finally, the protein bands were observed using enhanced chemiluminescent (ECL).

### 3.10. Gene Set Enrichment Analysis (GSEA)

GSEA was performed using GSEA 3.0 (http://www.broadinstitute.org/gsea/, accessed on 14 November 2021). Statistical significance and degree of enrichment were quantified by nominal *p*-value and normalized enrichment score (NES). The enrichment analysis of the Kyoto encyclopedia of genes and genomes pathways (KEGG) was performed by GSEA.

### 3.11. Antibiotics (Abx) Pretreatment

The antibiotics (Abx) consisted of vancomycin (50 mg/kg), ampicillin (100 mg/kg), metronidazole (100 mg/kg), and neomycin (100 mg/kg) mixed with sterile normal saline. Each mouse was given oral gavage of antibiotics (0.2 mL daily) 3 days before DSS administration.

### 3.12. Gut Microbiota Analysis

Gut microbiota composition was determined by 16S rRNA gene sequencing as described previously [26].

### 3.13. Transcriptome Analysis

Total RNA was isolated with TRIzol reagent. The concentration of RNA was assessed using a Nanodrop spectrophotometer (IMPLEN, Westlake Village, CA, USA). RNA integrity was determined by Agilent 2100 (Agilent Technologies, Santa Clara, CA, USA). The sample libraries were generated using NEBNext UltraTM RNA Library Prep Kit for Illumina (NEB, USA) according to the manufacturer’s recommendations. The library was sequenced using Illumina Hiseq 4000 platform, and paired-end 150 bp reads were generated. Star and Cufflinks software was used to align transcripts and make a quantitative analysis of all genes. DEGs analysis was performed using the DESeq R package (1.10.1). The expression level of each transcript was calculated by HTSeq software (v 0.6.0), according to the fragments per kilobase of the exon. Per million mapped reads (FPKM) value of 1 was set as the threshold for judging whether the gene was expressed.

### 3.14. Statistical Methods

Data were represented as means ± SEM. Statistical difference between the experimental group and control group was analyzed by the Student’s *t*-test using GraphPad Prism 8 (GraphPad Prism Software, USA). Correlation analysis was performed using Pearson correlation analysis in SPSS version 26 (IBM SPSS Statistics). *p* values <0.05 were considered statistically significant.

## 4. Discussion

In recent years, new options have emerged to treat UC through targeting gut microbiota [27]. Natural alkaloids can modulate the gut microbiota to restore the barrier function of the intestinal mucosa, thus relieving colonic inflammation in vivo and in vitro [28]. Researchers have endeavored to hunt economical, safe, and effective active alkaloids for treating UC. In our previous study, we had found that CEP could exert therapeutic effects on UC, and meanwhile regulate the composition of gut microbiota [25]. However, we did not delve into the interaction between CEP and gut microbiota. In the present study, we verified that CEP ameliorated colitis through modulating macrophage infiltration and gut microbiota-related ACOD1 expression.

Prior studies have noted the implication of macrophages in the development of UC [29,30]. Macrophages can have high plasticity that enables them to polarize [31]. Switching between M1 and M2 types, macrophages play a dual role in the inflammatory response and promote tissue healing and repair [32]. A study has shown that the level of M1-like macrophages increases and that of M2-type macrophages decreases in the mice with DSS-induced colitis [33]. LPS derived from gut microbiota is the main activator for macrophages. Under the stimulation of LPS, macrophages undergo metabolic reprogramming from oxidative phosphorylation to glycolysis; in this process, succinate, a TCA cycle intermediate metabolite, accumulates and induces the secretion of inflammatory factors [34]. In our study, we found that after CEP was used to challenge macrophages stimulated by LPS, the secretion of inflammatory factors in macrophages decreased. Furthermore, IHC staining showed a decrease in intestinal macrophage levels in the CEP group. The above results suggest that CEP exerts its therapeutic efficacy on UC via inhibiting the macrophage infiltration in colon tissue of WT mice.

ACOD1 expression can be induced under pro-inflammatory conditions [35]. Inducible ACOD1 has also been considered a key regulon in the metabolic reprogramming of macrophages [20]. In our study, we found that ACOD1 was one of the significant DEGs between the CEP-treated group and the DSS-induced group. PCR and WB showed that ACOD1 mRNA and protein levels were decreased in the CEP group, suggesting that CEP may regulate the expression of ACOD1 in activated macrophages under the condition of colitis. Studies have shown that the expression of ACOD1 is significantly up-regulated in macrophages upon LPS stimulation, which promotes the polarization of macrophages to M1 type [36,37]. M1-type macrophages can produce a large number of pro-inflammatory cytokines and chemokines, thus aggravating inflammation and tissue damage [38]. This is consistent with our experimental results. In macrophages in vitro, ACOD1 knockdown decreased the protein expression of TNF-a, IL-1B, and IL-6 inflammatory factors. Therefore, it can be speculated that ACOD1 may promote macrophage polarization towards the M1 type.

Functionally, ACOD1 encodes cis-aconitate decarboxylase, which catalyzes the decarboxylation of cis-aconitic acid, an intermediate product of the tricarboxylic acid cycle, to itaconic acid [39]. As the metabolite of ACOD1, itaconic acid (ITA) can be modified into two forms: dimethyl itaconate (DI) and 4-octyl itaconate (4-OI) [20]. There are marked functional differences between modified ITA and prototype ITA. Under in vitro culture, modified ITA, either DI or 4-OI, can suppress the synthesis of interferon-β (IFN-β), while the prototype ITA can significantly promote the secretion of IFN-β by macrophages. Contrary to modified ITA, the prototype ITA may have proinflammatory effects, resulting in succinate accumulation and tumor progression [40]. In our previous study, the prototype ITA aggravated DSS-induced colitis and increased the levels of pro-inflammatory cytokines TNF-α, IL-1β, and IL-6 secreted by macrophages in vitro [41]. Currently, no studies have resolved whether ACOD1, as a key gene of ITA synthesis, can promote intestinal inflammation. A study has reported that β-glucan significantly suppresses the expression of ACOD1 and the production of TIA, and restores oxidative phosphorylation in macrophages [42]. Otherwise, β-glucan can also ameliorate colitis by regulating gut microbiota [43]. However, ACOD1 expression in UC patients has never been reported. We analyzed the IBD database in iHMP through data mining and found that ACOD1 was highly expressed in the intestinal tissue of UC patients, and this expression was positively correlated with the degree of intestinal inflammation.

Gut microbiota dysbiosis drives the progression of UC [44]. CEP can enhance the therapeutic effect of cisplatin (CDDP) by regulating gut microbiota [45]. Our experiments have revealed that the protein level of ACOD1 expression in colon tissue decreases after antibiotic pretreatment, suggesting that the alteration in the gut microbiota may be related to the expression of ACOD1. To verify this suggestion, high-throughput sequencing was used to investigate the influence of CEP on gut microbiota. The results showed that CEP dramatically reduced the abundance of *Peptostreptococcaceae*, *Enterobacteriaceae*, *Escherichia-Shigella,* and *Romboutsia*, all of which had been significantly elevated in the DSS group. Typically, the abundances of *Peptostreptococcaceae* and *Enterobacteriaceae* increase in patients with UC [46,47]. Consistently, the proliferation of *Escherichia-Shigella*, a typical genus of *Enterobacteriaceae*, is a potential risk factor for the deterioration of UC [48]. Interestingly, the abundances of certain gut microbiota, such as *Peptostreptococcaceae*, *Enterobacteriaceae*, *Escherichia-Shigella,* and *Romboutsia*, were positively correlated with ACOD1 expression level, suggesting that CEP exploits gut microbiota to regulate ACOD1 expression in UC. Taken together, CEP may alleviate intestinal inflammation by reducing the abundance of pro-inflammatory intestinal microbiota.

Nevertheless, this study has certain limitations. First, more analytical tools should be adopted to analyze the relationship between gut microbiota and ACOD1. Currently, it is impossible to extract one specific bacterial genus in the gut microbiota, which hinders our clarification of which strain of bacterium is targeted by CEP. Additionally, ACOD1 knockout mice should be used to explore the causal relationship between ACOD1 expression and intestinal inflammation in future studies.

## 5. Conclusions

**Figure 6:**
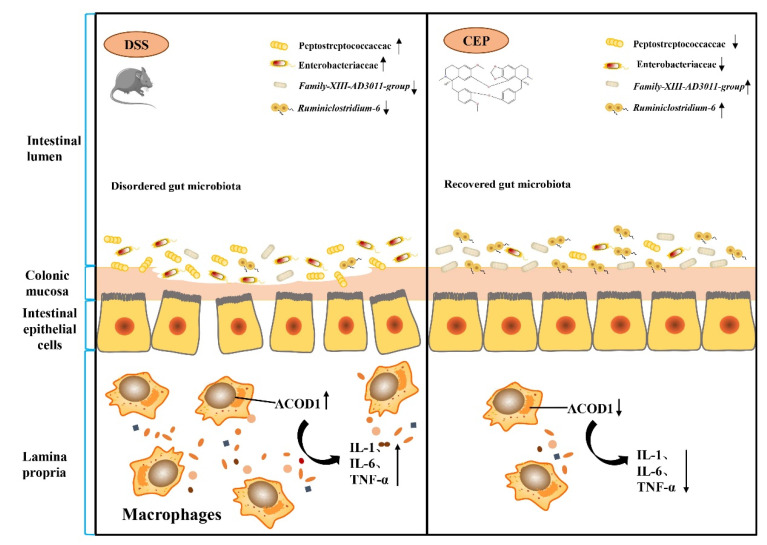
Illustration of the molecular mechanisms underlying the positive effect of CEP on experiment colitis. Cepharanthine restores the dysregulated gut microbiota to affect the macrophages infiltration and ACOD1 expression to improve the inflammation of colon tissue in colitis mice.

Our study conducted a preliminary mechanistic investigation of CEP to alleviate DSS-induced colitis. At the cellular level, CEP inhibits LPS-induced secretion of pro-inflammatory cytokines and ACOD1 expression in macrophages. In the mice with DSS-induced colitis in vivo, CEP represses macrophage infiltration and ACOD1 expression in the colon tissues. CEP also reduces the abundance of intestinal pro-inflammatory microbiota. CEP counters UC through modulating gut microbiota and inhibiting the expression of ACOD1 (Figure 6). CEP may serve as a potential pharmaceutical candidate in the treatment of UC.

## Figures and Tables

**Table 1:** DEGs between the CEP-treated group and the DSS group.

Gene_id	Gene	CEP	DSS	log2FoldChange	pval
ENSMUSG00000026180	Cxcr2	1.03334917	94.16767	−6.5098	3.17E-15
ENSMUSG00000056071	S100a9	3.600703256	591.049	−7.3589	2.51E-09
ENSMUSG00000025746	Il6	0.789597786	139.0612	−7.4604	9.42E-09
ENSMUSG00000022126	Acod1	1.819814268	129.1428	−6.149	1.70E-08
ENSMUSG00000056054	S100a8	3.893089097	352.1094	−6.499	3.17E-08
ENSMUSG00000026531	Mptx1	64050.71091	224.1921	8.1583	2.12E-06
ENSMUSG00000038067	Csf3	0.237174896	31.54608	−7.0554	2.62E-06
ENSMUSG00000026532	Spta1	176.3861308	0.870293	7.663	4.58E-06
ENSMUSG00000031722	Hp	19.52932338	1314.396	−6.0726	1.57E-05
ENSMUSG00000079180	Mptx2	103.2173205	0.816104	6.9827	6.47E-05

## Data Availability

Our data are available upon request for academic researchers.
